# The Effect of Low-Carbohydrate Diet on Macrovascular and Microvascular Endothelial Function Is Not Affected by the Provision of Caloric Restriction in Women with Obesity: A Randomized Study

**DOI:** 10.3390/nu12061649

**Published:** 2020-06-02

**Authors:** Chueh-Lung Hwang, Christine Ranieri, Mary R. Szczurek, Assem M. Ellythy, Ahmed Elokda, Abeer M. Mahmoud, Shane A. Phillips

**Affiliations:** 1 Department of Physical Therapy, University of Illinois at Chicago, Chicago, IL 60612, USA; clhwang@uic.edu (C.-L.H); christineranieri@gmail.com (C.R.); mszczurek89@gmail.com (M.R.S.); aellyt2@uic.edu (A.M.E.); amahmo4@uic.edu (A.M.M.); 2 Department of Rehabilitation Sciences, Florida Gulf Coast University, Fort Myers, FL 33965, USA; aelokda@fgcu.edu; 3 Department of Medicine, Division of Endocrinology, Diabetes, and Metabolism, University of Illinois at Chicago, Chicago, IL 60612, USA

**Keywords:** low-carbohydrate diet, hypocaloric, isocaloric, women health, obesity, conduit artery, microvasculature, nitric oxide, cardiovascular risks, primary prevention

## Abstract

Obesity impairs both macro- and microvascular endothelial function due to decreased bioavailability of nitric oxide. Current evidence on the effect of low-carbohydrate (LC) diet on endothelial function is conflicting and confounded by the provision of caloric restriction (CR). We tested the hypothesis that LC without CR diet, but not LC with CR diet, would improve macro- and microvascular endothelial function in women with obesity. Twenty-one healthy women with obesity (age: 33 ± 2 years, body mass index: 33.0 ± 0.6 kg/m^2^; mean ± SEM) were randomly assigned to receive either a LC diet (~10% carbohydrate calories) with CR (*n* = 12; 500 calorie/day deficit) or a LC diet without CR (*n* = 9) and completed the 6-week diet intervention. After the intervention, macrovascular endothelial function, measured as brachial artery flow-mediated dilation did not change (7.3 ± 0.9% to 8.0 ± 1.1%, *p* = 0.7). On the other hand, following the LC diet intervention, regardless of CR, blocking nitric oxide production decreased microvascular endothelial function, measured by arteriolar flow-induced dilation (*p* ≤ 0.02 for both diets) and the magnitude was more than baseline (*p* ≤ 0.04). These data suggest improved NO contributions following the intervention. In conclusion, a 6-week LC diet, regardless of CR, may improve microvascular, but not macrovascular endothelial function, via increasing bioavailability of nitric oxide in women with obesity.

## 1. Introduction

Over the past decades, obesity rates are increasing in the United States with more than 1 in 3 adults having obesity [1]. Obesity is associated with several adverse health conditions including hypertension, dyslipidemia, and hyperglycemia [2]. These metabolic abnormalities combined with obesity triple the risks of cardiovascular disease [3], which remains the leading cause of death in the United States [4]. The number of men and women with obesity are similar, but the prevalence of morbid obesity (body mass index, BMI ≥ 40 kg/m^2^) among women is reported to be almost twice as high as the prevalence among men [1]. Therefore, early intervention for young and middle-aged women is important to decrease the severity of obesity and prevent cardiovascular disease.

The critical site of arteriosclerosis development and the progression to cardiovascular disease is endothelium [5]. Endothelium is a single layer of endothelial cells that form the most inner layer of every blood vessel of the circulation. It synthesizes and releases several vasodilators and vasoconstrictors and plays a key role in controlling vascular tone and maintaining vascular homeostasis. Nitric oxide (NO) is a potent vasodilator synthesized by the endothelium. Decreases in NO bioavailability due to endothelial dysfunction causes impaired endothelium-dependent vasodilation [6]. Assessments of endothelium-dependent vasodilation in peripheral blood vessels have been widely used as surrogate markers of cardiovascular disease risks. The peripheral blood vessels refer to all blood vessels external to the heart and includes the macrovasculature (4 to 25 mm in diameter) and microvasculature (arterioles < 150 µm in diameter). The function and structure of these vasculatures are different: The macrovasculature buffers the increases in blood flow pulsatility, preventing tissue injury, and distributes blood to the body, whereas the microvasculature regulates vascular tone/resistance and blood pressure. In adults with obesity, endothelium-dependent vasodilation in both macro- and micro-vasculature are impaired [7,8].

A low-carbohydrates (LC) diet has been shown to improve glucose control in adults with obesity [9,10,11,12,13,14,15,16]. However, the effect of LC diet on endothelial function in adults with obesity is not clear with conflicting data, showing either no changes [9,10,12,13,14], increases [17], or even decreases in endothelial function following LC diet [11,15,16]. The conflicting results may be due to the heterogeneity in subject characteristics, LC diet interventions (e.g., % energy restriction, % carbohydrate restriction, fat content, and dietary sources), and experimental designs (e.g., single group or randomized controlled study). One of the potential confounders is the provision of caloric restriction (CR; 20–30% daily energy restriction or 400–800 kcal/day deficit). For studies examining the effect of CR diet without modifications in nutrition components, some found that CR improves both macrovascular and microvascular endothelial function [18,19,20], while others showed that CR does not have beneficial effects on macrovascular endothelial function [21,22,23]. In addition, many of the previous studies examining the effect of LC diet focused on macrovascular endothelial function. The effect of LC diet on microvascular endothelial function is lacking and may be different from macrovascular endothelial function [14,24].

Therefore, to isolate the effect of LC diet and its effect on different vasculatures, we conducted a randomized parallel design clinical trial in young and middle-aged women with obesity. We compared the effect of LC diet with vs. without CR on macro- and microvascular endothelial function. We hypothesized that LC without CR diet, but not LC with CR diet, would improve macro- and microvascular endothelial function in this population.

## 2. Materials and Methods 

### 2.1. Study Design

**Figure 1:**
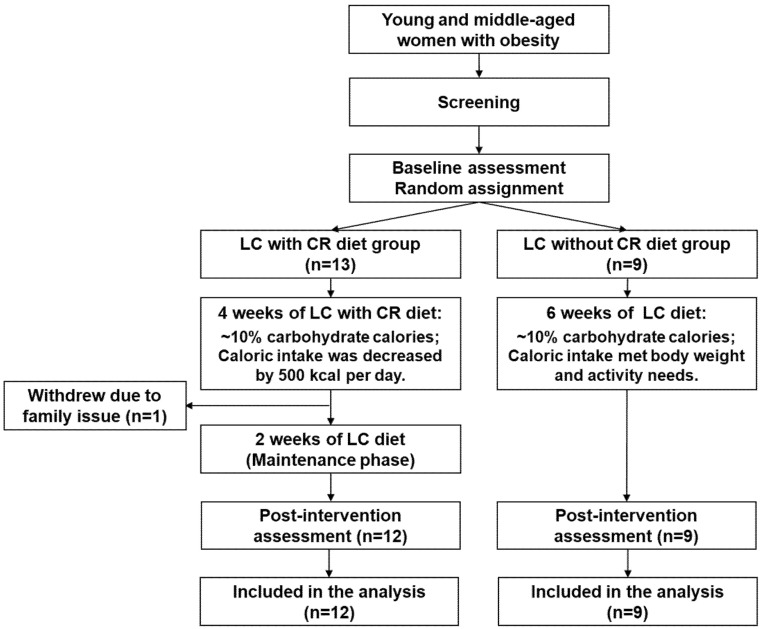
Study flow chart. CR = caloric restriction; LC = low-carbohydrate.

A prospective randomized parallel design clinical trial was conducted at the Clinical Research Center, at the University of Illinois at Chicago. Subjects were recruited from university campuses and local health clubs via notices posted on bulletin boards and in newsletters. Subjects were also recruited via Craigslist. After informed consent was obtained, participants completed a screening visit to determine qualification using physical examination, self-reported medical history, blood analysis including metabolic panel, lipid profile, insulin, and thyroid function test, and urine pregnancy test. Participants who met the inclusion criteria were enrolled by our research coordinator and were randomized to either a LC with CR diet or LC without CR diet (Figure 1). Macro- and microvascular endothelial function (primary outcomes) as well as cardiovascular risks (secondary outcomes) were assessed before and after the 6-week diet intervention. Data analyses were completed by researchers blinded to the participant diet assignment. Participants were instructed to continue their usual physical activity and received a pedometer (T5E011, Timex Group USA, Inc., Middlebury, CT, USA) to monitor their daily walking activity during the intervention. The average of 7-day step counts was calculated every 2 weeks. Two participants in LC without CR group did not have pedometer record. 

The study was approved by the institutional review board of the University of Illinois at Chicago and complied with the Declaration of Helsinki. Written informed consent was obtained from all study participants.

### 2.2. Study Participants

Apparently healthy women aged between 18 to 50 years, not currently on a diet, and with BMI of 29.0–39.9 kg/m^2^ were included in this study. Participants were excluded if they had any one of the following: (1) Inability to give informed consent; (2) no willingness to commit to the LC diet intervention; (3) history of cardiovascular disease or cardiovascular events, diabetes, renal or liver diseases; (4) hypertension (systolic > 160 mmHg and diastolic > 90 mmHg) or use of a antihypertensive drug; (5) history of head injury (past 6 months), seizure disorder, pituitary tumor or thyroid disease; (6) history of gout; (7) glaucoma or adverse reaction to nitroglycerin, lidocaine allergy, or anemia; (8) history of tobacco use (past 6 months), currently abusing alcohol or illicit drugs; (9) a diagnosis of eating disorder, current use of diet pills, history of diet (past 1 month), or current use of antioxidant supplements; (10) prior weight loss surgery (any type); (11) pregnancy (or intend to become pregnant while participating in trial), nursing, or amenorrhea.

### 2.3. Low-Carbohydrate Diet Intervention

All participants received a custom made individualized 6-week LC diet (10% carbohydrate, 60–62% fat, 28–30% protein). Daily caloric intake was determined by the Mifflin equation for women [25,26] as following: Planned daily caloric intake = Resting energy expenditure (REE) × Physical activity factor, where REE = (10 × body weight (kg)) + (6.25 × body height (cm)) – (5 × age (years)) − 161, and activity factor = 1.2 for sedentary (little or no exercise), 1.375 for lightly active (light exercise, 1–3 days/week), and 1.550 for moderately active (moderate exercise, 3–5 days/week). The factor was determined based on self-reported physical activity. For CR group, participants had a caloric deficit by 500 calories/day less than their calculated values during the first 4 weeks, and then the regular calculated values during the last 2 weeks of the study (Figure 1). The maintenance phase was designed in order to prevent further changes in body weight, which may confound the measurement of endothelial function when individuals are actively losing weight.

To ensure compliance to the diet, all participants were provided the meals for their appropriate diet regime for the entire intervention ranging from 40–51 days until the post-intervention assessments were completed. Meals were delivered to or picked up by participants, 3 times/week. Before the intervention, participants met with the bionutritionist to discuss the diet design. A three-day dietary record was used to determine their usual dietary patterns. In addition, a food preference questionnaire was completed to help plan the diet menus. All meals were prepared by the same bionutritionist, with moderate additions of salt, pepper, dried herbs, and spices for palatability. The major source of protein was from animals. Participants could consume unlimited water and zero-calorie beverages, however, with caffeinated coffee limited to 16 oz/day and diet soda and caffeinated tea limited to 20 oz/day. Participants also received daily multivitamin tablets.

Participants were asked to provide daily food diary and document any deviation from the provided meals. Any leftover food was returned and collected from the participants to ensure accuracy of calculating actual dietary intake. Every 2 weeks, participants met with the bionutritionist to discuss any problems (such as nausea, dizziness, constipation, lethargy, dehydration, bad breath, and loss of appetite) or concerns of adhering to the diet and to test their urine ketone levels. During the week 4, caloric intake was re-calculated for LC with CR diet group and diet modifications were discussed with participants for the final 2 weeks. Dietary intake and nutrients were analyzed using DietMaster Pro V11 (Lifestyles Technologies, Inc., Grants Pass, OR, USA).

### 2.4. Randomization

The table of random numbers was created by a statistician using a computer-generated random stratified sequence to ensure a balanced ethnic/racial composition within each group. Participants were assigned to the intervention by a staff member who did not know participant’s baseline profile.

### 2.5. Study Procedures

The following procedures were performed in the morning, after a 12-h fast with no caffeine, alcohol, and medication use. Participants were also instructed not to exercise at least 12 h prior to the visits.

#### 2.5.1. Macrovascular Endothelial Function

As previously described [27], flow-mediated dilation (FMD), a measure of endothelium-dependent vasodilation [28], was assessed non-invasively using brachial artery diameter in response to increases in blood flow followed by a transient period of ischemia. Briefly, participants rested in a supine position for at least 15 min, in a quiet, darken, and temperature-controlled room. Imaging of the brachial artery was taken via a 11mHz transducer and the MicroMaxx ultrasound machine (SonoSite, Seattle, WA, USA). The probe was placed ~5 cm above the antecubital fossa of the right arm, abducted ~80 away from the body. Blood flow velocity was determined via a continuous wave Doppler with an insonation of 60. After a 1-min baseline imaging period, Doppler readings of peak flow were recorded for at least 5 s. Then a blood pressure cuff placed on the forearm (distal to the antecubital fossa and right next to the antecubital crease) was inflated to >50 mmHg above supine systolic blood pressure for 5 min. After the cuff release, Doppler readings of peak flow were recorded for the first 10 s. Then, brachial artery was imaged continuously to capture diameter at 30 s, 1 min, 2 min, and 3 min after the cuff release. After 10 min following the release of the cuff, nitroglycerin (NTG)-mediated dilation, independent of endothelial function, was measured as previously described [27]. Briefly, imaging of the brachial artery was taken for 1 min before and for 5 min after the administration of 0.5 mg of NTG sublingually. Along with NTG-mediated dilation, FMD is the gold standard measure of macrovascular endothelial function. 

All images were digitally recorded and transferred to Brachial Imager (Medical Imaging, Iowa City, IA, USA) for analyzing the brachial artery diameter. For each baseline and time point, ~75 frames (7.5 frames per second for 10 s) were analyzed to calculate the average of the continuous diameters over the entire cardiac cycle. Both FMD and NTG-mediated dilation were calculated as the percentage of the maximal change after cuff release or NTG administration using the following equation: %change = (peak diameter − baseline diameter)/(baseline diameter) × 100%. The peak flow velocity was measured by analyzing 5 s for baseline and 10 s following the release of the cuff. Shear rate was calculated as blood velocity divided by brachial diameter. To assess NO bioavailability, serum nitrates and nitrites (NOx) levels, a surrogate marker of NO levels, were assessed using commercially available kits following the manufactory guideline (Cayman Chemicals, Ann Arbor, MI, USA).

#### 2.5.2. Microvascular Endothelial Function

As previously described [29], microvascular endothelial function was assessed using flow-induced dilation (FID) in arterioles isolated from subcutaneous adipose tissue of participants. Briefly, participants received subcutaneous fat biopsy with sterile techniques performed by a trained clinician. A small fat biopsy was obtained just underneath the skin of the gluteal region. The skin was locally anesthetized with lidocaine. A small incision (~1 cm) was made to expose the subcutaneous fat and approximately 1 mL of fat tissue was removed by sharp dissection. The incision was closed with Steristrips and covered with a waterproof clear bandage. The fat tissue then was transferred to the HEPES solution (pH= ~7.4 and 4 C). Due to the invasive nature of this procedure, we obtained both baseline and week 6-tissues in a total of 13 participants (LC without CR diet: *n* = 5 and LC with CR diet: *n* = 8).

Adipose arterioles were isolated from the fat tissue and cannulated with glass micropipettes in an organ perfusion chamber. The chamber was circulated with physiological salt solution (pH = ~7.4) using a peristaltic pump and bubbled with air (5% CO_2_ and 21% O_2_) at a temperature of 37 °C. Following a 30-min pressurization at 60 cm H_2_O, arterial diameter was measured at baseline, following a pre-constriction with endothelial-1 (100 to 200 pM), and during intraluminal flow corresponding to pressure gradients of Δ10–Δ100 cm H_2_O (5 min each) via an inverted microscope attached to a video monitor and a video-measuring device (model VIA-100; Boeckeler). Vessels were discarded when the pre-constriction with endothelial-1 was less than 30% of baseline diameter. FID at each pressure gradient was calculated as the percentage change using the following equation: %change = (diameter measured for each pressure gradient − pre-constricted diameter by endothelin-1)/(baseline diameter − pre-constricted diameter by endothelin-1) × 100%. At the end of each protocol, endothelium-independent vasodilation was induced by papaverine (10^−4^ M). This protocol was repeated in the presence of the endothelial NO synthase inhibitor (L-NAME, 10^−4^ M) to determine the contribution of NO in FID. 

#### 2.5.3. Cardiovascular Risks

Body composition was assessed using a Hologic QDR-4500 fan-beam DXA scanner (Hologic Inc., Bedford, MA, USA). Also measured, were body weight, height (for calculating BMI), and waist and hip circumferences. Venous blood samples were drawn from the arm by trained clinicians into serum tubes and ethylenediaminetetraacetic acid-containing tubes. Fasting blood lipids, insulin, and glucose were analyzed by Alverno Clinical Laboratories (Hammoond, IN, USA). The homeostasis model assessment of insulin resistance (HOMA-IR) was calculated to assess insulin resistance. Seated blood pressure was measured after a 5-min rest using a stethoscope and a blood pressure cuff with a sphygmomanometer. The assessment of cardiovascular risks was repeated at the end of week 4 (except 1 participant in LC with diet and 1 in LC without diet group did not receive blood draw).

### 2.6. Statistical Analysis

Data are presented as mean ± SEM or n (%). Statistical analyses were performed based on the original diet assignment using IBM SPSS Statistics (Version 24, Chicago, IL, USA). Statistical significance was set at α = 0.05. To examine baseline differences between groups (LC with CR diet vs. LC without CR diet), independent *t*-test and χ2 were used for continuous and categorical variables, respectively, except that between-group differences in baseline FID was examined using a two-way (2 × 5) mixed ANOVA with a between-subject factor (LC with CR diet vs. LC without CR diet) and a within-subject factor (pressure gradients of Δ10–Δ100 cm H_2_O). To examine dietary intake during the intervention between two groups, independent *t*-test was used. To examine physical activity changes between groups during the intervention, a two-way (2 × 4) mixed ANOVA was used with group as a between subject factor (LC with CR diet vs. LC without CR diet) and time as a within subject factor (baseline, 2 weeks, 4 weeks, and 6 weeks after the intervention). Main effect of time and group were examined separately if no interaction was found and Bonferroni post hoc pairwise comparisons were performed to examine physical activity between each time point in all participants. 

To examine the effect of the LC diet with vs. without CR on macrovascular function and cardiovascular risks, a two-way (2 × 2) mixed ANOVA was used with group as a between subject factor (LC with CR diet vs. LC without CR diet) and time as a within subject factor (baseline and 6 weeks after the intervention). For cardiovascular risks, the two-way mixed ANOVA was repeated with time as a within subject factor (baseline and 4 weeks after the intervention). When the interaction between the two factors was significant, then Bonferroni post hoc pairwise comparisons were performed. Main effect of time and group were examined separately if no interaction was found.

To examine whether FID changed after the 6-week of intervention within each group (LC without CR diet and LC with CR diet), main effect of time was examined by using a two-way (2 × 5) repeated-measures ANOVA with two within-subject factors: (1) Timing of measurement: baseline and 6 weeks after the intervention and (2) pressure gradients of Δ10–Δ100 cm H_2_O. To examine the effect of LNAME on FID at each timepoint (baseline and 6 weeks after the intervention) within each group, main effect of condition (presence or absence of LNAME) was examined by using a two-way repeated-measures ANOVA with two within-subject factors: (1) Presence or absence of LNAME and (2) pressure gradients of Δ10–Δ100 cm H_2_O.

## 3. Results

### 3.1. Dietary Intake and Physical Activity

A total of 21 women (age: 33 ± 2 years and BMI: 33.0 ± 0.6 Kg/m^2^) completed the intervention and were included in this study (Figure 1). One participant withdrew from the study due to a death in the family. Overall, participants enjoyed LC diet with good and normal appetite. No serious adverse event was observed. Participants in LC without CR group reported hunger (7.9%), bloating or too much food (3.5%), stomach problems (1.1%), no appetite (0.5%), and nausea (0.2%). In LC with CR group, participants reported hunger (5.9%), loss of appetite or too much food (1.1%), illness or nausea (0.9%), and stomach problems (0.2%). 

Caloric intake and dietary composition were not different between groups at baseline (mean for all participants: 2015 ± 101 kcal/day; 44.5 ± 1.4% from carbohydrate, 35.3 ± 2.2% from fat, and 18.4 ± 1.3% from protein; *p* ≥ 0.4; Table 1). During the intervention, actual caloric intake in LC without CR group was not different from the baseline value (*p* = 0.4) but less than the planned value (*p* = 0.007; Table 1). The latter caused a lower compliance in LC without CR group vs. LC with CR group (Table 1). However, LC without CR group still consumed more calories than LC with CR group during the 6-week intervention (*p* = 0.03) and no difference was found in the percent energy from carbohydrate, fat, and protein (*p* ≥ 0.1; Table 1). Positive urine ketone levels were noted in seven out of the 21 participants at the end of week 2, eight participants at the week 4, and six participants at the week 6 (*p* ≥ 0.2 for comparing the number of participants between LC with CR diet vs. LC without CR diet). 

Compared to the baseline, physical activity, measured by steps per day, significantly increased in all participants at the end of week 2 and week 4 (*p* = 0.01 for both; Table 1). At the end of week 6, although physical activity was not statistically significant compared to baseline (*p* = 0.1; Table 1), five out of seven participants in LC without CR group (data missing in two participants) and eight out of 12 participants in LC with CR group showed an increase in their daily steps ranging from 368 to 6964 steps/day. 

### 3.2. Participant Characteristics and Baseline Values 

**Figure 2:**
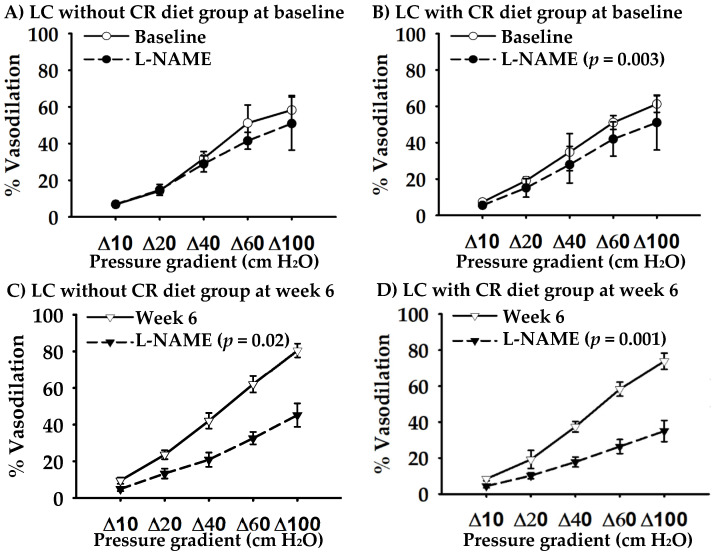
Arteriolar flow-induced dilation at baseline and after the 6-week low-carbohydrate (LC) diet intervention without caloric restriction (CR) diet (*n* = 5) and with CR diet (*n* = 8). Endothelial nitric oxide synthase inhibitor (L-NAME) was used to determine the contribution of nitric oxide in vasodilation.

No baseline differences were found between LC without CR and LC with CR groups in participant characteristics and cardiovascular risks (*p* ≥ 0.1; Table 2). Baseline FMD, NTG-mediated dilation, and FID were not different between the two groups (*p* ≥ 0.1; Table 3 and Figure 2A,B). L-NAME did not change baseline FID in LC without CR group (*p* = 0.4; Figure 2A). In LC with CR group, L-NAME decreased overall baseline FID by 6% (*p* = 0.003; Figure 2B).

### 3.3. Effect of LC Diet on Macro- and Micro-Vascular Endothelial Function

After 6 weeks of the intervention, FMD and NTG-mediated dilation remained unchanged (*p* ≥ 0.4 for time effect; Table 3). Serum nitrate/nitrite levels did not change following the intervention (14.2 ± 2.0 to 15.5 ± 2.9 µmol; *p* = 0.7 for time effect). 

In response to LC without CR diet, overall FID at week 6 significantly increased by 11% vs. baseline (*p* = 0.01 for the time effect). L-NAME decreased the overall FID at week 6 by 20% (Figure 2C) and this change was higher than baseline (*p* = 0.04). In response to LC with CR diet, although FID did not change (*p* = 0.1), L-NAME decreased overall FID at week 6 by 19% (Figure 2D) and this change was higher than baseline (*p* = 0.007).

### 3.4. Effect of LC Diet on Cardiovascular Risks

At the end of week 4, a significant interaction was noted only in body weight (*p* = 0.049; other outcomes: *p* ≥ 0.1). Post hoc pairwise comparisons indicated that body weight significant decreased by 2.6 kg in LC without CR diet (*p* = 0.001) and 3.4 kg in LC with CR diet (*p* < 0.0005; *p* = 0.4 for between-group comparison; Table 2). In addition, BMI and blood triglyceride levels significantly decreased in response to the LC diet regardless of CR (*p* ≤ 0.009 for time effect; Table 2). Other outcomes remained unchanged (*p* ≥ 0.1 for time effect; Table 2).

After 6 weeks of the intervention, body weight, BMI and % body fat significantly decreased (*p* ≤ 0.004 for time effect; *p* ≥ 0.8 for interaction effect; Table 2). Diastolic blood pressure and blood triglyceride levels also significantly decreased in response to the LC diet regardless of CR (*p* ≤ 0.04 for time effect; *p* ≥ 0.5 for interaction effect; Table 2). There was a significant interaction in blood high-density lipoprotein cholesterol levels (*p* = 0.03), however, with no significant post hoc pairwise comparisons noted (*p* ≥ 0.08; Table 2). Other outcomes remained unchanged (*p* ≥ 0.06 for time effect; *p* ≥ 0.2 for interaction effect; Table 2). No significant group effects were found in any outcomes (*p* ≥ 0.4).

## 4. Discussion

This randomized trial is the first study to examine whether the effect of LC diet on both macro- and micro-vascular endothelial function is influenced by the provision of CR in young and middle-aged women with obesity. Our major findings are: (1) Six weeks of LC diet did not change macrovascular endothelial function, measured as brachial artery FMD, regardless of CR; and (2) LC diet without CR increased microvascular endothelial function, measured by arteriolar FID, while following the LC diet, regardless of CR, the magnitude of FID decreased by L-NAME was more than baseline. These findings suggest that the effects of LC diet on macro- and micro-vascular endothelial function are different and the latter benefits from LC diet by improving NO contribution to vasodilation. Furthermore, LC diet decreased cardiovascular risks in women with obesity, as evident by decreases in body weight, BMI, % body fat, diastolic blood pressure, and blood triglyceride levels after the intervention. 

Brachial artery FMD reflects the nature of conduit artery or macrovascular endothelial biology. Following a transient period of ischemia (inflating a pressure cuff on the forearm for 5 min, and then deflating it rapidly), blood flow as well as shear stress increase [27]. The latter is considered as a physiological stimulus for inducing the release of NO from endothelium to vascular smooth muscle cells, thus causing vasodilation [27]. Brachial artery FMD is also an independent predictor of cardiovascular disease. For every 1% decrease in FMD, there is a 13% increase in the risks of future cardiovascular events [30]. A recent meta-analysis reported that obesity is associated with a 2% decrease in FMD [7], which may contribute to a 26% increase in cardiovascular disease risks. Therefore, brachial artery FMD is an important therapeutic target in adults with obesity to prevent cardiovascular disease. Previous studies have examined the effect of LC diet on FMD but their findings are either confounded by the provision of CR [9,10,12,13,14,15,16,17] or based on a single group design [31]. We found that the 6-week LC diet, regardless of CR, did not change FMD in women with obesity. In addition, we found that shear stress (measured as peak shear rate), systemic NO bioavailability (measured as serum NOx levels), and brachial artery smooth muscle function (measured as NTG-mediated dilation) remained unchanged following the intervention. Collectively, these findings suggest that LC diet, at least short-term (6 weeks), does not have effects on macrovascular endothelial function in young and middle-aged women with obesity. 

In agreement with our findings, previous studies demonstrated no change [9,10,12,13,14] or even a decrease [11,15,16] in FMD following a LC diet in adults with obesity. On the other hand, one study found that LC diet improved FMD [17]. The heterogeneity in subject characteristics, LC diet interventions (e.g., % energy restriction, % carbohydrate restriction, nutrient component, and dietary sources), and experimental designs among the published studies makes the comparisons difficult to explain the conflicting results. One factor noted in all three studies showing the deleterious effect of LC diet on FMD is the use of a very low carbohydrate diet combined with CR (≤30 g/day or 4–5% caloric intake from carbohydrate + 25–30% of CR) [11,15,16]. Such modes of LC diets may cause an insufficient intake of important micronutrients such as folate and dietary fiber [11,16], both of which have been shown to provide protective effects on endothelial health [32,33,34]. In our study, fiber intake was different between the two LC diet groups. However, we did not see significant correlations between the changes in dietary fiber and the changes in endothelial function (data not reported). To our knowledge, only one study demonstrated improvements in FMD following a LC diet [17]. This study included subjects with elevated triglycerides (mean: 211 ± 58 mg/dL). In our study and other studies showing no positive effects on FMD, subjects had lower levels of triglycerides (baseline mean: 72–151 mg/dL) [9,10,11,12,13,14,15,16]. Therefore, LC diet may have benefits on FMD only when macrovascular endothelial dysfunction is associated with elevated triglycerides. Our study and previous studies found that LC diet decreased blood triglycerides [9,10,14,15,17].

Microvascular endothelial function is vital for regulating peripheral vascular tone/resistance and blood pressure. In response to shear stress, the vascular endothelium generates and releases NO, causing vasodilation. Endothelial NO synthase is an enzyme responsible for NO production. In the current study, we measured FID in arterioles isolated from gluteal fat tissues to assess microvascular endothelium-dependent vasodilation. In addition, to determine the contribution of NO in FID, we applied L-NAME, endothelial NO synthase inhibitor, to the arterioles and blocked NO production. At baseline, we found that LNAME decreased overall FID in LC with CR group, but not in LC without CR group. These findings suggest that FID in LC with CR group was NO dependent at baseline, while in LC without CR group, other pathways such as hydrogen peroxide may be responsible for the vasodilation [35]. The different vasodilatory mechanisms between the two groups at baseline may contribute to the different FID responses following the intervention. However, regardless of CR, we found that the 6-week LC diet improved NO contribution or bioavailability in the microcirculation. We did not measure oxidative stress and inflammation, both of which are known as the underlying mechanisms of reduced NO bioavailability. On the other hand, enhanced vasoconstriction, e.g., due to increased generation of cyclo-oxygenase (COX-1)-derived vasoconstrictor metabolites, may also contribute to impaired FID [36,37]. Future studies to further dissect the mechanisms by which LC diet improves microvascular endothelial function in obesity appear warranted.

Our findings suggest that the effect of LC diet may not be the same across the arterial tree. Consistent with this possibility, previous studies demonstrated that a 6-week LC diet (11% caloric intake from carbohydrate) did not change macrovascular but increased microvascular endothelial function [14]. The underlying mechanisms by which LC diet improves microvascular but not macrovascular endothelial function remain to be determined. One possible mechanism is related to the changes in adipose/fat biology or adipokines. Adipokines, such as adiponectin, leptin, resistin, and tumor necrosis factor-alpha, are secreted by fat tissue. Excessive fat, or obesity, is associated with a decrease in adiponectin and/or an increase in leptin, resistin, and tumor necrosis factor-alpha [38]. These changes in adipokines associated with obesity can lead to reduced bioavailability of NO and endothelial dysfunction [38,39,40]. We isolated arterioles from the subcutaneous adipose tissue of participants. Therefore, such type of arterioles, compared to the brachial artery, may have a direct and immediate effect of changes in adipokine profiles. Previous studies demonstrated that LC diet increased adiponectin [10,41] and decreased leptin [16]. Although we did not measure adipokines in the current study, % body fat decreased following the 6-week LC diet, which may contribute to the improvements in adipokine profiles. On the other hand, interventions longer than 6 weeks may be required to induce adaptations in macrovascular endothelial function.

Following the 6-week LC diet, body weight and BMI decreased in all participants with no differences between the two diet groups and that is unexpected. We defined CR diet as a caloric deficit by 500 calories/day less than the planned caloric intake. We calculated the planned caloric intake for each participant using the Mifflin equation, which considers individual body weight, height, age, and physical activity levels, and has been used in different diet interventions [26,37]. The planned caloric intake should match individual metabolic needs, be similar as their baseline caloric intake, and not induce any body weight changes. In LC without CR group, the planned/calculated caloric intake was higher than the baseline and actual caloric intake during the intervention. We provided meals to study participants, asked them to complete daily food dairy (including their appetite and any comments), and collected any leftover food from them to ensure accuracy of actual dietary intake during the intervention. We cannot exclude the following two possibilities: (1) The baseline caloric intake was underestimated by using a self-reported food diary, and (2) the planned caloric intake was overestimated by using the Mifflin equation due to self-reported physical activity factor. However, LC without CR group consumed overall calories more than LC with CR group during the 6-week intervention. Higher CR with longer intervention length may be required to induce more changes in body weight, other cardiovascular risk factors, and endothelial function. In addition, the maintenance phase in LC with CR diet may contribute to the non-significant differences between the two groups. Physical activity may influence our findings. Surprisingly, in our study, physical activity, measured as step counts per day from pedometer, increased similarly in all participants. The use of a pedometer to monitor physical activity is feasible and user-friendly. In addition, pedometers provide participants instant feedback and reinforcement of physical activity. However, we did not obtain data regarding physical activity duration, intensity, and type other than walking. Future studies can measure physical activity by using accelerometers along with physical activity logs. 

Several strengths and limitations are noted in our study. We employed a randomized parallel design to isolate the effect of LC diet from CR. We excluded subjects who had no willingness to commit to the LC diet intervention. We provided meals with daily monitoring and regular meeting with bionutritionist. Therefore, our study has high compliance (~90% or higher) and low dropout (*n* = 1 unrelated to the study) and we were able to control dietary intake at every meal. In addition, we did not see any serious adverse events and the most common problem reported by the participants is hunger (6–8%), which is expected in diet interventions. Overall, the LC diet was well-tolerated. Although we did not report the reasons and number of subjects who were screened but did not meet inclusion/exclusion criteria, our study design and diet intervention may impact the recruitment rate, leading to a small and uneven sample size for each group. A block randomization can be used to ensure equal number of subjects assigned to each diet group. We assessed both macro- and microvascular endothelial function to provide comprehensive evidence of the vascular effects of LC diet. None of our participants were taking oral contraceptives. Two participants in LC with CR group had a diagnosis of polycystic ovary syndrome. When we excluded the two participants from analysis, similar results were observed. Due to the intervention length (6 weeks), we did not control the menstrual cycle phase between baseline and post-intervention visits. For brachial artery FMD, we were not able to measure flow velocity and diameter simultaneously and this was a limitation of our technology. Instead of using an in vivo measurement of microvascular function, an ex vivo model of arterioles allowed us to dissect the mechanisms of changes in NO contributions following the LC diet, without the presence of neurohormonal factors. 

We focused on healthy young and middle-aged women with obesity. The severity of obesity is higher in women than men. Therefore, early intervention for women with obesity is essential to prevent the progression of obesity and the development of cardiovascular disease. Our findings may not be generalizable to men, other age groups, and disease populations, and even healthy individuals with normal body weight. In healthy young men whose BMI less than 30 kg/m^2^, a one-week LC without CR diet (~10% caloric intake from carbohydrate) significantly decreased brachial artery FMD [31]. However, our findings still provide clinically important implications. Our LC diet was well-tolerated and improved bioavailability of NO in microvasculature, and thus microvascular endothelial function in women with obesity. Microvascular endothelial function drives the development of several cardiovascular diseases including hypertension [42]. Therefore, a long-term LC diet may be beneficial in controlling blood pressure and decreases risks for cardiovascular disease in women with obesity.

## 5. Conclusions

In conclusion, our findings suggest that the effect of LC diet on macro- and micro-vascular function is not influenced by the provision of CR in young and middle-aged women with obesity. A 6-week LC diet, regardless of CR, may improve microvascular, but not macrovascular, endothelial function via increased bioavailability of NO. Our findings may provide clinical implications of LC diet to decrease risks of cardiovascular disease associated with obesity.

## Figures and Tables

**Table 1:** Dietary intake and physical activity at baseline and during the 6-week low carbohydrate diet intervention.

	LC without CR Diet (*n* = 9)	LC with CR Diet (*n* = 12)	P Between-Groups
**Baseline dietary intake**			
Caloric intake, kcal/day	1993 ± 109	2032 ± 161	0.9
Carbohydrate, %kcal	45.8 ± 2.4	43.5 ± 1.7	0.4
Fat, %kcal	33.7 ± 3.4	36.6 ± 2.9	0.5
Protein, %kcal	18.3 ± 2.5	18.5 ± 1.3	0.95
**Planned/provided diet**			
Caloric intake (6 weeks), kcal/day	2328 ± 129	1782 ± 42	<0.0005
Caloric intake (Week 1–4), kcal/day	-	1616 ± 46	-
Caloric intake (Week 5–6), kcal/day	-	2163 ± 57	-
Carbohydrate, %kcal	10		-
Fat, %kcal	60–62		-
Protein, %kcal	28–30		-
**Actual dietary intake**			
Caloric intake (6 weeks), kcal/day	2090 ± 132	1724 ± 43	0.03
Caloric intake (Week 1–4), kcal/day	-	1596 ± 59	-
Caloric intake (Week 5–6), kcal/day	-	2054 ± 55	-
Compliance, %	89.9 ± 2.9	96.8 ± 1.7 *	0.04
Carbohydrate, %kcal	10.3 ± 0.3	10.9 ± 0.4	0.5
Fat, %kcal	60.4 ± 0.3	59.9 ± 0.3	0.3
Protein, %kcal	29.3 ± 2.8	28.7 ± 0.3	0.1
Dietary fiber, g	24.0 ± 1.7	19.4 ± 1.0 *	0.03
Folate/Folic acid, mcg	141.2 ± 10.7	122.9 ± 7.0	0.2
Vitamin C, mg	80.2 ± 4.8	78.2 ± 3.5	0.8
Sodium, mg	3292 ± 195	2924 ± 86	0.1
Potassium, mg	2184 ± 342	2101 ± 226	0.8
**Physical activity-step counts**			0.3
Baseline, steps/day	5924 ± 813	5587 ± 702	
Week 2, steps/day	8787 ± 1002	6552 ± 797	
Week 4, steps/day	8622 ± 1251	6943 ± 855	
Week 6, steps/day	7887 ± 1342	6868 ± 835	

Data are mean ± SEM. CR = calories restriction; LC = low carbohydrate. * *p* < 0.05 vs. LC without CR diet.

**Table 2:** Participant characteristics and cardiovascular risks in response to the 6-week low carbohydrate diet intervention.

	LC without CR Diet (*n* = 9)			LC with CR Diet (*n* = 12)		
	Baseline	Week 4	Week 6	Baseline	Week 4	Week 6
Age, year	33 ± 3			32 ± 2		
Race						
Caucasian, *n*	4 (44)			5 (42)		
African American, *n*	3 (33)			4 (33)		
Hispanic, *n*	1 (11)			2 (17)		
Asian, *n*	1 (11)			1 (8)		
Body weight, kg ^b^	89.1 ± 4.6	85.7 ± 5.2 *	85.6 ± 4.5	90.0 ± 3.8	86.5 ± 3.9 *	87.5 ± 4.3
BMI, kg/m^2^ ^a,b^	33.5 ± 1.0	32.8 ± 1.1	32.3 ± 0.9	32.6 ± 0.8	31.2 ± 0.9	31.7 ± 0.9
Waist circumference, cm	96.1 ± 3.0	92.7 ± 3.3	92.2 ± 2.9	95.5 ± 2.7	93.6 ± 3.1	93.1 ± 3.2
Waist-to-hip ratio	0.88 ± 0.07	0.84 ± 0.05	0.84 ± 0.04	0.85 ± 0.02	0.81 ± 0.02	0.79 ± 0.03
Body fat, % ^b^	44.5 ± 0.7	44.1 ± 1.3	43.4 ± 0.9	43.7 ± 0.9	43.9 ± 1.1	42.5 ± 1.0
SBP, mmHg	115 ± 2	113 ± 3	115 ± 4	118 ± 5	112 ± 4	112 ± 3
DBP, mmHg ^b^	70 ± 1	68 ± 3	68 ± 2	72 ± 4	70 ± 3	67 ± 3
Total cholesterol, mg/dL	180 ± 14	194 ± 15	190 ± 14	185 ± 7	181 ± 8	182 ± 7
LDL cholesterol, mg/dL	106 ± 10	121 ± 12	117 ± 12	104 ± 7	110 ± 8	110 ± 7
HDL cholesterol, mg/dL	51 ± 4	56 ± 3	55 ± 3	60 ± 4	56 ± 3	58 ± 3
Triglycerides, mg/Dl ^a,b^	116 ± 23	92 ± 15	85 ± 13	104 ± 21	72 ± 6	71 ± 6
Glucose, mg/dL	89 ± 3	93 ± 3	91 ± 4	93 ± 4	89 ± 4	87 ± 3
Insulin, μU/mL	13.9 ± 2.6	12.5 ± 2.4	12.3 ± 1.7	13.5 ± 2.4	10.2 ± 2.2	11.8 ± 3
HOMA-IR	3.0 ± 0.6	3.0 ± 0.6	2.8 ± 0.4	3.2 ± 0.7	2.4 ± 0.6	2.6 ± 0.7

Data are mean ± SEM or *n* (%). BMI = body mass index; CR = calories restriction; DBP = diastolic blood pressure; HDL = high-density lipoprotein; HOMA-IR = homeostatic model assessment for insulin resistance; LC = low-carbohydrate; LDL = low-density lipoprotein; SBP = systolic blood pressure; * *p* < 0.05 vs. baseline (based on the post-hoc comparison test); ^a^ *p* < 0.05 for time effect (baseline vs. week 4) in all participants; ^b^ *p* < 0.05 for time effect (baseline vs. week 6) in all participants.

**Table 3:** Macrovascular endothelial function in response to the 6-week low carbohydrate diet intervention.

	LC without CR Diet (*n* = 9)		LC with CR Diet (*n* = 12)		P Group × Time	P Group	P Time
Brachial Artery	Baseline	Week 6	Baseline	Week 6			
FMD, %	6.5 ± 1.1	5.9 ± 1.5	7.8 ± 1.4	9.6 ± 1.6	0.4	0.1	0.7
Baseline diameter, mm	3.05 ± 0.17	3.15 ± 0.25	3.28 ± 0.15	3.24 ± 0.12	0.4	0.5	0.7
Maximum diameter, mm	3.26 ± 0.19	3.32 ± 0.24	3.52 ± 0.14	3.56 ± 0.14	0.8	0.3	0.4
Peak flow, cm/s	105 ± 12	120 ± 12	94 ± 10	106 ± 10	0.8	0.4	0.08
Peak shear rate,	334 ± 42	373 ± 40	269 ± 27	300 ± 29	0.9	0.1	0.2
NTG-mediated dilation, %	25.8 ± 3.8	24.7 ± 1.9	24.6 ± 2.5	28.5 ± 2.3	0.9	0.99	0.4
Baseline diameter, mm	3.01 ± 0.17	3.01 ± 0.20	3.34 ± 0.14	3.26 ± 0.14	0.6	0.2	0.6
Maximum diameter, mm	3.83 ± 0.21	3.76 ± 0.21	4.11 ± 0.13	4.16 ± 0.13	0.2	0.2	0.8

Data are mean ± SEM. CR = calories restriction; FMD = flow-mediated dilation; LC = low-carbohydrate; NTG = nitroglycerin.
